# Role of health-related quality of life measures in the routine care of people with multiple sclerosis

**DOI:** 10.1186/1477-7525-3-16

**Published:** 2005-03-18

**Authors:** Alessandra Solari

**Affiliations:** 1 Epidemiology Unit, National Neurological Institute C. Besta, Via Celoria 11, 20133 Milan, Italy

**Keywords:** Multiple sclerosis, health-related quality of life, outcomes assessment, clinical practice

## Abstract

Health-related quality of life instruments are expected to be of particular value in routine care of people with multiple sclerosis (MS), where they may facilitate the detection of disease aspects that would otherwise go unrecognised, help clinicians appreciate patient priorities particularly in terms of treatment goals, facilitate physician-patient communication, and promote shared decision-making. However, it appears that these instruments are little used routine clinical approaches to people with MS. To address this issue, I performed a bibliographic search of studies that evaluated the efficacy of generic or disease-specific health-related quality of life (HRQOL) instruments in MS clinical practice from clinicians' or patients' perspectives. I found only one cross-sectional study, which compared preferences for three instruments, and assessed acceptability in people with MS.

Reasons for lack of transfer of HRQOL measurements to clinical practice may be cultural, methodological, or practical. With regard to MS, the proliferation of instruments seems to constitute a barrier, with no particular instrument having gained wide popularity or consensus. Other barriers are lack of resources for the administration, collection and storage of the data, and inability of clinicians to score, interpret, and use HRQOL instrument to guide clinical care. It is therefore important to refine existing tools, extending clinical validation to wider contexts and cultures. More studies assessing acceptability and clinicians' and patients' preferences for different instruments are also required.

## Review

Multiple sclerosis (MS) is a demyelinating disease of the central nervous system of unknown etiology and poorly understood pathogenesis. There is a north-south gradient of MS prevalence in the northern hemisphere, with highest levels (over 100 per 100,000) in northern regions [1,2]. It is a chronic disease with a modest effect on life expectancy, but a broad spectrum of consequences, of variable severity, on physical and psychological characteristics, that vary between individuals and within individuals over time. The disease typically strikes women (2:1) in their peak years of career development and family life; commonly there are exacerbations and remissions followed by progression whose rate and extent vary [3]. There is also a benign form of MS, characterised by few relapses, long periods of remission, and mild activity limitations over the long term [4]. The available treatments have at best a modest benefit on the course of the disease [5].

### Health-related quality of life measures

Interest in measuring outcomes in MS has increased markedly over the past 20 years. Standardised instruments have been developed, the most-used being the Expanded Disability Status Scale (EDSS) [6] which is a mixed impairment/activity limitations scale based on neurological examination of eight functional systems, plus ambulation/mobility status. Despite major limitations – bias towards locomotor function, variable sensitivity to change according to scale score, and suboptimal inter-rater reliability – the EDSS is widely-used by researchers and clinicians because its scores are readily understood by all.

More recently, the importance of MS outcome assessment from the perspective of the person with the disease has been recognised [7]. After 1992, the number of publications on health-related quality of life (HRQOL) increased steadily, as did those employing MS-specific instruments (see Figure). Generic instruments were applied to MS [7-12], and disease-specific instruments were devised and validated [13-24]. The seven available MS-specific HRQOL instruments are listed in the Table 1; all were published between 1995 and 2001. Three consist of a generic module (SF-36 [13,15] or FACT-G [14]) plus an MS-specific module. In most cases people with MS participated in their development [13,16]. Except for the MS Quality of Life 54 (MSQOL-54), which has been translated into several languages [13,20-23], and the Functional Assessment of MS (FAMS), which is also available in Portuguese [24], these questionnaires are available only their original versions. Aspects of responsiveness were evaluated in four of the seven instruments, but in general sensitivity to change has been insufficiently investigated [18,25-28]

**Table 1:** Characteristics of MS-specific HRQOL questionnaires

	**MSQOL-54**	**FAMS**	**MSQLI**	**RAYS**	**HAQUAMS**	**MSIS-29**	**LMSQoL**
Publication year	1995	1996	1999	2000	2001	2001	2001
Generic module	SF-36 (36 items)	FACT-G (28 items)	SF-36 (36 items)	--	--	--	--
MS module	18 items	31 items	9 scales	50 items	38 items	29 items	8 items
People with MS involved in development	No	Yes	--	No	Yes	Yes	Yes
Versions	US English [13] Italian [20] French [21] Canadian French [22] Japanese [23]	US English [14] Portuguese [24]	US English [15]	Hebrew [16]	German [17]	English [18]	English [19]
Reliability	Alpha Test-retest [13,20–23]	Alpha [14,24]	Alpha Test-retest [15]	Alpha [16]	Alpha Test-retest [17]	Alpha Test-retest [18]	Alpha Test-retest [19]
Responsiveness	RCT [25] RCT [26]	--	RCT [27]	--	RCT [28]	Effect size [18]	--
Domains not assessed	Vision	Vision Bladder/ bowel Sexual function	--	--	--	Vision Sexual function	Vision
Time period assessed	Past 4 weeks Current time	Past week	--	Past week	Past year Past 4 weeks Past week	Past 2 weeks	Past month
Time to complete	20 minutes	20 minutes	--	--	20 minutes	--	--
Publications (no.)	16	10	5	1	3	7	2
Publication period	1995–2004	1996–2004	1999–2003	2000	2001–2004	2001–2004	2001

MSQOL-54 is the MS quality-of-life 54; FAMS is the Functional Assessment of MS; MSQLI is the MS Quality of Life; HAQUAMS is the Hamburg Quality of Life Questionnaire in MS; FACT-G is the Functional Assessment of Cancer Therapy, General version; LMSQoL is the Leeds MS Quality of Life. Alpha is Cronbach's coefficient alpha. RCT aspects of responsiveness assessed in randomized controlled trial.

### HRQOL and routine clinical practice

HRQOL studies in MS have drawn attention to the multiplicity of domains that may be compromised by the disease, and the effects of this compromise on ability to cope. As expected, people with MS, especially those with a progressive course, report reduced physical functioning compared to the general population [10,11,29-31]; they are more likely to suffer fatigue [29,32] and depression [32,33] than the general population, and are also more likely to be unemployed [8,10,30,31,34,35]. Unexpectedly, however, it has been reported that the importance attached to compromise in different HRQOL domains may vary considerably between MS sufferers and their neurologists [7].

The ultimate aim of measuring HRQOL is to provide a comprehensive assessment of patients' health status, to serve as a baseline from which to tailor interventions, pharmacological or otherwise, and assess their effectiveness, both in the clinical trial setting and in routine care. HRQOL instruments are expected to be of particular value in routine care, where they may improve the detection of disease aspects that would otherwise go unrecognised, help clinicians appreciate patient priorities particularly in terms of treatment goals, facilitate physician-patient communication, and promote shared decision making. In addition HRQOL data from clinical trials can provide information that clinicians can usefully discuss with their patients [36]. Unfortunately, although recent MS trials include some HRQOL assessment, there is no internationally agreed gold standard for conducting such assessment or reporting outcomes. HRQOL evaluations are not required as endpoints in MS trials by the European Agency for the Evaluation of Medicinal Products [37]. Even when HRQOL endpoints are included, data collection and reporting are often of poor quality [38] with the consequence that cost effectiveness issues, which HRQOL instruments can throw light on, such as preserved function, less work missed, and improved emotional well-being, are not analysed.

### Literature survey

It appears that HRQOL instruments are little used in routine clinical approaches to people with MS. To address this issue, I searched MEDLINE (1966–2004), the Cochrane Library (Issue 1, 2005) and the Cochrane MS Group trials register (2004) for studies that evaluated the efficacy of generic or MS-specific HRQOL instruments in clinical practice from the clinicians' or MS patients' perspective, also checking study references. Studies considering patient-reported outcomes other than HRQOL, and domain-specific measures were excluded.

I found only one study, a cross-sectional postal survey conducted in Canada, published in 2004 [39]. This study assessed MS sufferers' preferences regarding two generic instruments (the EuroQol EQ-5D and the SF-36), and an MS-specific instrument (MSQOL-54). Over 90% of 183 participants reported that EuroQol EQ-5D and the SF-36 were acceptable or very acceptable, and 85% did so for MSQOL-54. Surprisingly, over 75% of participants felt that a combination of the three instruments best described their HRQOL.

The reasons for lack of transfer of HRQOL assessment into clinical practice may be cultural, practical, or methodological [40-43]. With regard to cultural factors, patients generally welcome the opportunity to provide clinicians with information regarding their HRQOL [43]. That this is also the case for people with MS is suggested by high participation rates in most postal surveys assessing patient-reported health status [30,35,39,44], and by the good acceptability of HRQOL instruments [39]. By contrast, information on practicing clinicians' perceptions of the utility of HRQOL data is limited and conflicting: studies have uncovered a lack of knowledge of HRQOL as well as concerns that these instruments may be a covert means of assessing physicians' performance [45,46].

Practical considerations be particularly important in clinical settings, where data must be provided promptly and in an understandable manner to be of use. Instruments must be administered, processed, scored, stored and retrieved – all of which have logistic and financial implications [47]. Most HRQOL instruments are lengthy and may be burdensome for patients and clinicians. For most existing instruments, the score is not immediately available, but needs to be calculated, while score interpretation may not be straightforward. For example a recently published study on transplant physicians found that 55% would be more likely to use HRQOL data if it were more comprehensible [48]. In the United States time spent gathering and interpreting HRQOL information as part of the clinical encounter is not built into reimbursement by third-party payers [49]. It is noteworthy, however, that questionnaire length seems not to be a drawback for people with MS since a combination of HRQOL instruments was preferred by over 75% of participants in the only study found [39].

Another factor limiting the dissemination of HRQOL tools in MS clinical practice is likely to be that too many instruments are available, and unlike EDSS, none has emerged as clearly superior to any other.

## Conclusion

Existing HRQOL tools for people with MS should be refined and their clinical validation pursued in the widest possible cultural context. More studies assessing instrument acceptability and preferences of clinicians and people with MS are also needed. It would be useful for example to implement computer-based technology (touch-screens and adaptive administration to reduce respondent burden by selecting pertinent items and omitting inappropriate ones) and other alternatives to traditional paper-and-pencil or interview methods, which should of course be evaluated for acceptability and reliability [48]. The objective is not to add HRQOL measurements to the chores of everyday practice, but to incorporate meaningful HRQOL instruments into the care process [50].

**Figure 1:**
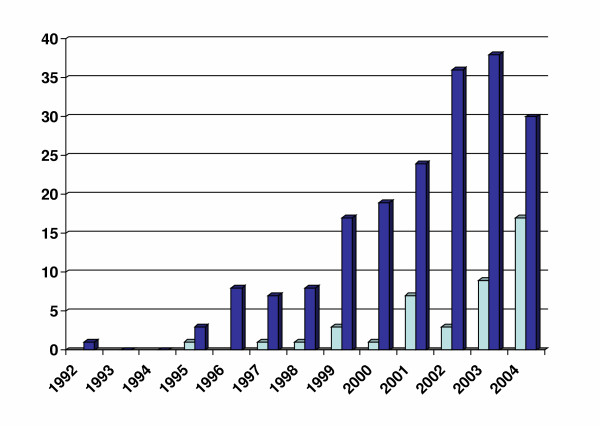
Number of publications on HRQOL in people with MS between 1992 and 2004. Blue bars indicate all studies on HRQOL; light blue bars indicate studies employing MS-specific instruments. Studies considering patient-reported outcomes other than HRQOL, or domain-specific measures are excluded.
